# Proteoglycan Sulphation in the Function of the Mature Central Nervous System

**DOI:** 10.3389/fnint.2022.895493

**Published:** 2022-05-30

**Authors:** James W. Fawcett, Jessica C. F. Kwok

**Affiliations:** ^1^Department of Clinical Neurosciences, John van Geest Centre for Brain Repair, University of Cambridge, Cambridge, United Kingdom; ^2^Centre for Reconstructive Neuroscience, Institute for Experimental Medicine Czech Academy of Science (CAS), Prague, Czechia; ^3^Faculty of Biological Sciences, School of Biomedical Sciences, University of Leeds, Leeds, United Kingdom

**Keywords:** chondroitin sulphate, heparan sulphate, perineuronal net, memory, plasticity, neuroregeneration, neurodegeneration, stem cells

## Abstract

Chondroitin sulphate and heparan sulphate proteoglycans (CSPGS and HSPGs) are found throughout the central nervous system (CNS). CSPGs are ubiquitous in the diffuse extracellular matrix (ECM) between cells and are a major component of perineuronal nets (PNNs), the condensed ECM present around some neurons. HSPGs are more associated with the surface of neurons and glia, with synapses and in the PNNs. Both CSPGs and HSPGs consist of a protein core to which are attached repeating disaccharide chains modified by sulphation at various positions. The sequence of sulphation gives the chains a unique structure and local charge density. These sulphation codes govern the binding properties and biological effects of the proteoglycans. CSPGs are sulphated along their length, the main forms being 6- and 4-sulphated. In general, the chondroitin 4-sulphates are inhibitory to cell attachment and migration, while chondroitin 6-sulphates are more permissive. HSPGs tend to be sulphated in isolated motifs with un-sulphated regions in between. The sulphation patterns of HS motifs and of CS glycan chains govern their binding to the PTPsigma receptor and binding of many effector molecules to the proteoglycans, such as growth factors, morphogens, and molecules involved in neurodegenerative disease. Sulphation patterns change as a result of injury, inflammation and ageing. For CSPGs, attention has focussed on PNNs and their role in the control of plasticity and memory, and on the soluble CSPGs upregulated in glial scar tissue that can inhibit axon regeneration. HSPGs have key roles in development, regulating cell migration and axon growth. In the adult CNS, they have been associated with tau aggregation and amyloid-beta processing, synaptogenesis, growth factor signalling and as a component of the stem cell niche. These functions of CSPGs and HSPGs are strongly influenced by the pattern of sulphation of the glycan chains, the sulphation code. This review focuses on these sulphation patterns and their effects on the function of the mature CNS.

## Introduction

All the cells of the central nervous system (CNS) are surrounded by a diffuse extracellular matrix (ECM), and around some classes of neurons ECM is condensed into a compact pericellular ECM structure called perineuronal nets (PNNs). The ECM in the CNS has a wide range of functions, most of them mediated by proteoglycans, which consist of a core protein to which are attached a number of chains of glycosaminoglycans (GAGs). The GAG chains can be sulphated to a varying extent and in different positions to give various charge structures as chondroitin sulphate proteoglycans (CSPGs) and heparan sulphate proteoglycans (HSPGs). The binding properties and functions of these chondroitin sulphate and heparan sulphate GAG (CS-GAG, HS-GAG) chains are largely determined by their sulphations, both in terms of charge and the resulting structure. Sulphation patterns change with development, ageing, inflammation and different forms of damage. This review addresses the effects of these patterns of sulphation on a range of CNS functions.

### Synthesis and Degradation of Chondroitin Sulphate and Heparan Sulphate

**FIGURE 1:**
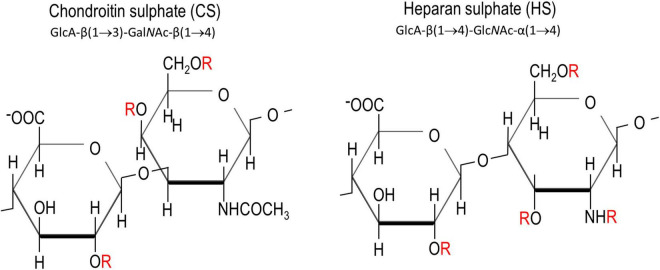
Chemical structures of chondroitin sulphate and heparan sulphate. “R” in red denotes the potential sites for sulphations. These are C2 on glucuronic acid, C4 and C6 on N-acetyl galactosamine, and C3, C6 and the amine group on N-acetyl glucosamamine.

Chondroitin sulphates and heparan sulphates are two major members of GAGs. They are both sulphated polysaccharides made up of repeating disaccharides of hexuronates and hexosamines (Figure 1). While CS-GAG is derived from the polymerisation of glucuronic acid (GlcA) and N-acetylgalactosamine (Gal*N*Ac) units, HS-GAG is derived from GlcA and N-acetylgluosamine (Glc*N*Ac). Epimerisation of the carboxyl group by C-5 epimerase produces an epimer of GlcA called the iduronic acid (IdoA), providing one fundamental modification for CS- and HS-GAGs. Sulphation in the disaccharide unit presents another common modification for GAG chains. This could happen at carbon (C)-4, C-6 of Gal*N*Ac (in CSPGs), C-2, C-3, C-6 acetyl group of Glc*N*Ac (in HSPGs), C-2 in GlcA (in CSPGs and HSPGs) (Figure 1). While sulphation of GAGs in CS is usually spread evenly along the GAG chains, sulphation in HS usually occurs as clusters creating local sulphated and non-sulphated domains, along the chain (Guglieri et al., 2008; Kwok et al., 2012). Heparin, utilised for its anti-coagulation activity, is a highly sulphated form of HS.

The addition of GAG chains to core proteins takes place in the Golgi apparatus where the addition of a tetrasaccharide linkage (-xylose-galactose-galactose-GlcA-) at the serine residue on the core protein signals for the synthesis of CS and HS chains. Monosaccharides are then added to the linkage region one by one in an alternating fashion from the concerted action of Gal*N*Ac transferases, Glc*N*Ac transferases, GlcA transferases, chondroitin synthases, CS polymerising factors or EXT1 and 2 (Sarrazin et al., 2011; Kwok et al., 2012). Sulphation occurs as the nascent GAG chains are being polymerised by the sulfotransferases present at the luminal membrane of the Golgi apparatus (Kitagawa et al., 1997b). In mammals, sulphated CSs are produced by the family of carbohydarate sulfotransferases (Chsts) while sulphated HSs are produced by the family of heparan sulfotransferases (Hssts and Ndsts) (Sugahara and Kitagawa, 2000; Esko and Lindahl, 2001).

The main route for removal of CS- and HS-GAGs is catabolism under acidic pH in the lysosomes where glycosidases, hydrolases and sulfatases act to digest the glycosidic linkage and remove the sulphate group (Litjens and Hopwood, 2001). Recently extracellular glycosidases (e.g., Hyal4) or sulfatases (e.g., Arsb) have been reported which function at physiological pH and can degrade GAGs in the ECM (Zhang et al., 2014; Kowalewski et al., 2021).

The pattern of sulphation along HS and CS GAG chains creates local charge domains and specific structures that can define binding sites of varying specificity for a variety of ligands. For instance C-4, 6-sulphated CS-GAG (also called CS-E) binds strongly to the Nogo receptor and in some situations to semaphorin 3A (Dickendesher et al., 2012; Dick et al., 2013; Mikami and Kitagawa, 2013).

### Chondroitin Sulphate Proteoglycans Sulphation in Normal Physiology, Injury, and Ageing

**FIGURE 2:**
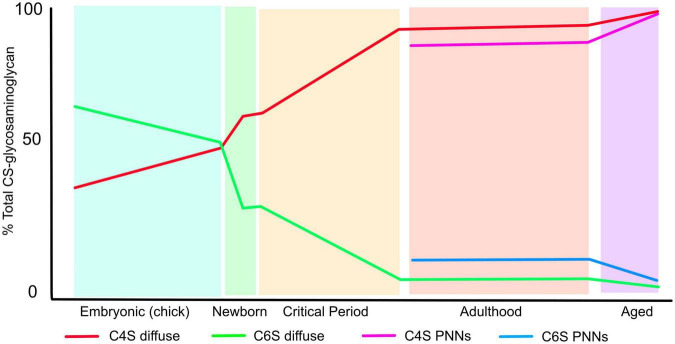
The pattern of CS-GAG sulphation changes during the lifespan. The graph shows the percentage of CS-GAG decorated with C4S and C6S during development, postnatal, critical periods for plasticity, adulthood and ageing. The figures for embryonic development come from chick embryos brains, the rest from rat brains (see text for references). The figure shows the progressive loss of C6S and increase in C4S during the lifetime. The sulphation pattern of PNNs differs from that of the diffuse ECM.

The CNS ECM changes greatly during development and ageing. For instance during embryogenesis the overall relative volume of ECM compared to cells is much greater than later in life, and later in development PNNs appear during the postnatal period. During these changes in composition and condensation, the pattern of sulphation of CSPGs also changes (Figure 2). In early embryogenesis the predominant form of CS-GAG is chondroitin 6-sulfate (C6S) which makes up 60% of the total, chondroitin 4-sulphate (C4S) is 30%, disulphated forms are barely detectable the remainder being unsulphated. As embryogenesis progresses C6S progressively decreases and C4S increases, so in pre-hatching chicks both make up 43% of the total. The disulphated forms C2, 6S and C4, 6S increase to 5 and 2%, respectively (Kitagawa et al., 1997a). Postnatal rat brain ECM contains 18% C6S, 59% C4S, 2% C2, 6S and C4, 6S and 19% unsulphated (Carulli et al., 2010). This composition changes little until the critical periods for plasticity. After this point the adult pattern of sulphation is observed, and it now differs between the diffuse brain ECM and the PNNs. C6S is 2% in the diffuse ECM and 5% in PNNs, C4S is 91% in diffuse ECM, 81% in PNNs and C4, 6S is 1% in diffuse ECM, 2% in PNNs. There are also small differences between HSPGs in diffuse matrix and PNNs, with overall sulphation being 0.54 per disaccharide in diffuse matrix against 0.67 per disaccharide in PNNs (Deepa et al., 2006). The final changes come in the aged brain, where the quantity of C6S reduced to 0.6% and almost all the ECM is 4-sulphated (Foscarin et al., 2017). It is also worth noting that the ratio of CS vs. HS is 9–1 in the adult CNS.

In the adult CNS, CSPGs are present mainly in the diffuse ECM and on PNNs. Their inhibitory role in axon regeneration is exemplified by the upregulation of multiple CSPGs around sites of injury, where the CSPGs are present in and around lesions and on glial cells (Fawcett and Asher, 1999). For memory and plasticity, the CSPGs in PNNs are the main effector. Thus digestion of CS-GAGs with chondroitinase ABC affects CSPGs in the diffuse matrix and PNNs, and has effects on axon regeneration, plasticity and memory. Genetic deletion of link protein *Crtl1* or *aggrecan* in the CNS affects PNNs with little effect on the diffuse matrix, yet has the same effects on plasticity and memory as chondroitinase ABC (Carulli et al., 2010; Romberg et al., 2013). PNNs are found mainly around parvalbumin positive GABAergic inhibitory neurons, which are known to play a central part in the regulation of memory and plasticity.

These changes in sulphation pattern are significant for axon regeneration, sprouting and plasticity as described below. In general, all forms of CS-GAG are inhibitory to the growth of both PNS and CNS axons *in vitro* to a certain extent, although the response depends on the type and developmental stage of the neurons: for instance in embryonic hippocampal neurons, contactin on the neuronal surface can interact with C4, 6S (CS-E) to promote outgrowth (Mikami et al., 2009), the phosphacan/DSD-1 can promote or inhibit outgrowth depending on the neuronal lineage and developmental stage (Garwood et al., 1999) and C2, 6S, which is present in low amounts in mammals, can promote neurite outgrowth by interaction with αvβ3 integrin (Shida et al., 2019). However, C4S exerts stronger inhibition to neurite growth than C6S. The inhibition of neurite outgrowth by C6S is overcome by small amounts of a positive growth signal such as laminin, while C4S is inhibitory even in the presence of high laminin concentrations. Thus as the CNS matures and finally ages, the ECM becomes progressively more inhibitory as the ratio of C4S to C6S progressively increases (Figure 2). In the aged brain, C6S in PNNs becomes almost undetectable and PNN matrix is therefore highly inhibitory, with consequences to memory as described later (Foscarin et al., 2017). As described below, these changes have important effects on CNS function. Damage and inflammation also cause changes in many molecules of the ECM.

The HSPGs glypican and syndecan are found in PNNs, synapses and other matrix structures in the CNS, and astrocyte-released glypicans participate in the formation of excitatory synapses (Allen et al., 2012). The role of HSPG sulphation in synaptogenesis has received less attention. However, 3-O-sulphated glypican is necessary for normal synapse formation in the *C. elegans* mating circuit (Lazaro-Pena et al., 2018), and a blocker of 3-sulphated HS impaired synapse assembly in hippocampal neurons *in vitro* (Maiza et al., 2020).

Damage to the CNS leads to a strong local increase in the quantity of CSPGs around the injury site, multiple CSPGs being upregulated (Fawcett and Asher, 1999). With the increased production of core proteins comes changes in the sulphation pattern of their CS-GAGs. The upregulation of CSPGs around injuries, optic nerve damage, strokes, and inflammation has been demonstrated by immunostaining, western blotting and mRNA assays in many studies. Around the injury the staining is usually associated with an apparent barrier at which regenerating axons stop. In general CNS damage is associated with an increase in C4S and there may also be some increase in C6S (Properzi et al., 2005). There is a matching increase in expression of mRNA for C4ST-1 in glial cells (Properzi et al., 2005; Wang et al., 2008; Yi et al., 2012; Pearson et al., 2020). There are also changes in sulfotransferases for HSPGs (Properzi et al., 2008).

Sulphation motifs can be detected by antibodies that recognise their charge and structure. A series of monoclonal antibodies that recognise PNNs was raised by the Hockfield lab, each of which recognises a subset of PNNs. One of these, Cat-315 is expressed before PNN formation in extrasynaptic sites prior to synapse formation, and at this time decorates an O-mannose linked epitope on the CSPG, phosphacan/PTPζ. Later in development, in PNNs, the antibodies recognise aggrecan, and staining was absent in aggrecan knockouts and after chondroitinase ABC treatment, identifying the binding sites as glycoforms of aggrecan (Lander et al., 1998; Matthews et al., 2002). A well-characterised glyco-motif is DSD-1 defined by binding to the 473HD monoclonal antibody, found on phosphacan/PTPζ, and particularly associated with dividing CNS progenitor populations.

### Plasticity

PNNs around parvalbumin positive interneurons play a key role in the control of plasticity. The ending of critical periods for plasticity corresponds with PNN development, and critical period closure can be prevented and reversed by digestion of PNNs with chondroitinase or genetic attenuation of PNNs (Pizzorusso et al., 2002; Carulli et al., 2010). There are large changes in the sulphation of CSPGs in the CNS around the critical periods with decreased C6S, increased C4S (see above and Figure 2). Replacement of the C6S by transgenic overexpression of C6 sulphotransferase *Chst3* led to animals with persistent ocular dominance plasticity while deletion of *Chst3* produced mice with abnormally low levels of CNS plasticity after peripheral nerve regeneration (Lin et al., 2011; Miyata et al., 2012).

### Memory

A link between the ECM and memory is well-established, and most of the effect of ECM on memory is *via* PNNs. Memory is a specialised form of plasticity, and a link between the ECM and control of plasticity has been known for many years (see below). There was therefore a search for a link between ECM properties and memory. Most of the evidence linking ECM and memory came from digestion of CS-GAGs with chondroitinase ABC which digests all glycoforms of CS-GAGs, and affects several types of memory including fear, object recognition, social, place and substance addiction (Sorg et al., 2016). In most of these models, digestion of CSPGs enhanced memory acquisition or retention, or increased the extinguisability of long-term memories. Similar effects are seen in animals with deletions of either link protein Crtl1 or aggrecan which attenuate PNNs with little or no effect on the general interstitial ECM, indicating that it is the CSPGs in PNNs rather the interstitial ECM that have the strongest effects on memory (Romberg et al., 2013; Rowlands et al., 2018). Since C4S is responsible for most of the inhibitory properties of adult CSPGs and PNNs, it might be expected that neutralising this inhibition would affect memory. This was tested for object recognition memory, which depends on perihinal cortex (PrC). Chondroitinase digestion of PrC leads to a prolongation of object recognition memory. A blocking antibody (Cat316) to C4S was validated for its ability to neutralise PNN glycans to inhibit neurite outgrowth. This antibody injected into PrC produced a prolongation of object recognition memory identical to that seen after chondroitinase treatment. This suggests that PNNs modulate memory through their content of C4S (Yang et al., 2017). In adulthood sulphation levels in the undamaged CNS are stable, but with advanced age C6S in PNNs is drastically reduced, making PNN ECM extremely inhibitory to neurite growth (Foscarin et al., 2017). An obvious hypothesis was that these increasingly inhibitory PNNs might play a part in memory loss in ageing. This was tested in spontaneous object recognition (SOR) memory, which again depends on the PrC. The link was confirmed by restoring permissive C6S to PrC by viral vector or transgenic delivery of chondroitin 6-sulphotransferase *Chst3* (Yang et al., 2021). Sulphotransferase-treated mice showed no memory loss with ageing, and an increase in the ratio of inhibitory to excitatory synapses on parbalbumin positive interneurons. In contrast, transgenic animals lacking *Chst3* from birth showed memory loss at a very early age. However, transgenic animals with reduced expression chondroitin-4-sulphotransferase-1 (*Chst11*), the main enzyme that produces C4S, perform similarly to C6S overexpressers, and show only minimal loss of object and place memory with advancing age and a similar change in synapses on parvalbumin positive neurons (Ruzicka et al., 2021). The implication is that the balance between C6S and C4S in PNNs determines the inhibitory nature of PNNs. If C6S is lost, PNNs become inhibitory with increasing difficulty in making new synapses, thus inhibiting new memory formation. The concept was used to treat memory loss in transgenic mouse line expressing mutant tau as a tauopathy model of Alzheimer’s disease. Here the Cat316 monoclonal antibody mentioned above that selectively binds to C4S and blocks its inhibitory effects was used. Treatment of the tauopathic mice at an age when they showed complete loss of SOR memory restored memory function (Yang et al., 2017). While 4-sulphate CS-GAGs inhibit memory as described above, 4-sulphation of dermatan sulphates has the opposite effect. Knockout of *Chst14/D4st1*, which adds 4-sulphate to dermatan, led to impaired spatial learning and memory, and reduced hippocampal long term potentiation.

### Axonal Regeneration

Damage to the CNS or PNS leads to a glial reaction with increased local production of several CSPGs by astrocytes, oligodendrocyte precursors, pericytes, and meningeal cells (Fawcett and Asher, 1999). Since most CSPGs are inhibitory to axon growth in *in vitro* models, an implication is that the CSPGs in glial scar tissue may participate in the blockage of axon regeneration in the CNS, and also in regeneration failure in PNS injuries where there is extensive scarring. A large number of experiments using chondroitinase ABC to digest the CS-GAG chains have supported this general concept, with increased local axon regeneration and sprouting in regions of chondroitinase digestion (Burnside et al., 2018; Rosenzweig et al., 2019). However, reactive astrocytes can be permissive as well as inhibitory (Anderson et al., 2018). CSPGs can affect regeneration directly through binding to the PTPsigma or LARS receptors (Lang et al., 2015), Nogo receptor (Dickendesher et al., 2012), or through presentation of some of the many molecules such as Semaphorin 3A that bind to the highly charged CS-GAG chains (Dick et al., 2013; Miller and Hsieh-Wilson, 2015). Does the sulphation of the CS-GAGs affect regeneration? In general C4S GAGs have proven to be highly inhibitory both in *in vitro* assays and *in vivo.* It appears to be the ratio of inhibitory C4S to permissive C6S that determines the inhibitory properties of the ECM. Thus using *in vitro* assays the inhibitory nature of C4S and permissive nature of C6S can be shown (Wang et al., 2008), and since C4S is upregulated after injury (Wang et al., 2008; Yi et al., 2012), overcoming its inhibition is an obvious target. The positive effect of C6S was shown in regeneration and plasticity experiments in a transgenic mouse with deletion or over-expression of the C6-sulfotranferase-1 (*Chst3*) gene. This codes for the enzyme responsible for most C6S synthesis and animals that do not express it have very low C6S levels. Regeneration was assayed in the mouse nigro-striatal pathway, which has a considerable amount of spontaneous regeneration after injury which can be increased by chondroitinase treatment. In the *Chst3* knockout animals there was almost no spontaneous regeneration, implying that the ECM which now contained almost all C4S was highly inhibitory (Lin et al., 2011). On the contrary, transgenic mouse with over-expression of *Chst3* demonstrated persistent plasticity in the adult CNS (Miyata et al., 2012). Experiments targeting removal of C4S have used the enzyme arylsulfatase B (ARSB) which cleaves 4-sulphated from the reducing end of CS-GAG chains. Treatment with ARSB was successful at enabling axon regeneration after optic nerve crush and spinal cord injury, with some function recovery in the spinal injury model (Yoo et al., 2013; Pearson et al., 2018). Knockouts of sulphation enzymes have produced mixed results. Knockout of C4-Sulphotransferase-1 (*Chst11*), the main 4-sulphation enzyme, in zebrafish led to accelerated axon regeneration after spinal injury. Interestingly, knockdown of dermatan-sulphate-4-sulforatransferase 1 (*Chst14*) had no effect (Sahu et al., 2019), while in mice the *Chst14* knockout reduced regeneration. C4, 6 di-sulphated CS-GAG is one of the three disulphated forms of CS present at around 2% of total CS-GAG, and it binds with high affinity to several ligands, including the PTPsigma CSPG/HSPG receptor, and to several trophic factors and to semaphorin 3A (Brown et al., 2012; Dick et al., 2013; Katagiri et al., 2018; Sakamoto et al., 2019). A blocking antibody to this sulphation form enabled axon regeneration in the optic nerve (Brown et al., 2012). The PTPsigma receptor is an important modulator of regeneration, binding to both CSPGs and HSPGs. The two types of proteoglycan have opposite effect *via* this receptor, with CSPGs inhibiting axon growth while HSPGs are growth promoting. Regulation of autophagy flux is a downstream effect of the receptor. Screening synthetic oligomers and octosaccharides with different sulphation patterns revealed that C4, 6S is the binding motif for CSPGS, while most sulphated HS oligomers bind to the receptor (Sakamoto et al., 2019).

The role of HSPG sulphation in axon regeneration is less clear. A variety of syndecans and glypicans are present on the neuronal surface, and have been implicated in embryonic axon growth and synaptogenesis. The inhibitory action of part of the Nogo-A molecule on axon growth is HS-GAG dependent (Kempf et al., 2017). Knockout of Sulf1 and Sulf2 enzymes that remove some 6-O-sulphate groups from HS-GAG led to decreased embryonic axon growth (Kalus et al., 2015). In a search for treatments to modulate astrogliosis and to improve regeneration after CNS damage, a number of heparin mimetics were characterised, with the finding that low-sulphated heparins were permissive, high sulphated heparins inhibitory (McCanney et al., 2019). The balance between HS-GAG and CS-GAG binding to the PTPsigma receptor determines its effects on axon regeneration. Examination of a series of HS octasaccharides indicated that any HS with at least one sulphate would bind and cancel the inhibitory effect of C4, 6S and permit neurite outgrowth (Sakamoto et al., 2019).

Overall, the ratio of C4S to C6S determines the degree of inhibition of the ECM to neurite extension. Interventions that increase the effect or amount of C6S or decrease those of C4S will enhance axon regeneration.

### Neurodegeneration

Proteoglycans are involved in several aspects of neurodegenerative disease. First they can participate in the degenerative process by promoting protein aggregation, internalisation into neurons and prion-like spread. Then, as PNNs, they can have a neuroprotective function and regulate plasticity and memory.

Aggregation of tau filaments, amyloid-β (Aβ) and α-synuclein is promoted by HSPGS. For tau protein, 6-O and 2-O sulphated HS can promote aggregation, while it is the extent rather than the position of sulphation that influences HS-mediated aggregation of Aβ and α-synuclein. Aβ aggregation is particularly associated with the HS-GAG chains on perlecan and agrin (Mah et al., 2021). For internalisation of tau, which is involved in the prion-like spread of tau pathology, 6-O and 3-O sulphation is a driver (Rauch et al., 2018), with syndecan the probable core protein. High expression of the enzyme that mediates 3-O sulphation, 3-O HS sulphotransferase (Hs3st1), is a risk factor for Alzheimer’s disease (Ennemoser et al., 2022). HSPGs are also involved in the clearance of amyloid from the brain (Morawski et al., 2014). Production of Aβ is influenced by HSPGs, with 6-O sulphation being an inhibitor of the APP-cleaving enzyme BACE (Schworer et al., 2013). Alzheimer’s disease also affects the HS-GAGs produced by the brain, with HS-GAGs extracted from affected brains having an increased ability to aggregate tau, and decreased binding to several important CNS growth factors, including BDNF (Huynh et al., 2019).

The proteoglycan-containing PNNs play a part the progress of Alzheimer’s disease, and in the ability of the CNS to accommodate to the advancing disease and preserve memory function. An initial observation was that PNN-bearing neurons in Alzheimer’s brains are seldom affected by tau pathology, and removing PNNs allows tau aggregates to form (Morawski et al., 2010; Suttkus et al., 2016). The PNNs themselves are neuroprotective against oxidative stress (Suttkus et al., 2012; Steullet et al., 2017). Disulphated C4, 6S CS-GAG is protective against excitatory amino acid toxicity (Sato et al., 2008), so could be involved in PNN neuroprotection.

Alzheimer’s disease affects many neurons scattered throughout the CNS. Maintenance of CNS function must depend on plasticity within the CNS, allowing new circuitry to grow so that damaged neurons can be bypassed. Removal of PNNs, or reducing their inhibitory properties both enhance plasticity and restore memory loss in ageing (Carulli et al., 2016; Yang et al., 2021). This strategy was used to treat transgenic mice expressing a mutant form of tau, and a transgenic mouse expressing mutant APP, both of which are models of Alzheimer’s disease. Digestion of PNNs with chondroitinase restored memory in both models, but more relevant to the topic of this review, as described above, blocking inhibitory C4S the CAT316 with a monoclonal antibody injected in the PrC restored object recognition memory in tauopathy mice with advanced disease (Vegh et al., 2014; Yang et al., 2014, 2017).

### Chondroitin Sulphate and Heparan Sulphate in Neural Stem Cells

In addition to the influence on axonal growth and sprouting, CS-GAGs and HS-GAGs also have a role in the regulation of stem cell proliferation and differentiation (Yamaguchi, 2001; Ida et al., 2006). During CNS development, CS sulphation changes from 6-sulphated CS being the major form in the early embryo and 4 sulphated CS predominating at the end of embryogenesis (Figure 2; Kitagawa et al., 1997a; Sugahara et al., 2003).

One of the most studied CSPGs in neurogenesis is NG2. While the expression of NG2 has often been associated with glial-progenitors such as oligodendrocyte progenitor cells and astrocytes (Levine and Card, 1987; Chang et al., 2000), studies have shown that NG2 positive cells possess the ability to differentiate into all CNS cell lineages, including neurons (Kondo and Raff, 2000; Dayer et al., 2005). The differentiation appears to be mediated through fibroblast growth factor 2 (FGF2), which can bind to both CS- and HS GAGs (Ida et al., 2006; Guglieri et al., 2008). FGF2 has previously been shown to bind HS-GAGs in a sulphation specific manner, and form a ternary complex with FGFR1, inducing cell proliferation and differentiation (Nurcombe et al., 1993; Schlessinger et al., 2000; Guglieri et al., 2008). FGF2 binding to CS-GAGs, on the other hand, requires highly sulphated CSs such as C6S, C2, 6S, and C4, 6S (Ida et al., 2006).

DSD-1 is a CSPG sulphation motif which regulates neurogenesis. The DSD-1 epitope (also called Mab-473 HD epitopes) is a CS structure which is found around neurogenic niches, including the germinal layers during forebrain development and the subependymal zone of the lateral ventricle in the adult brain (von Holst et al., 2006; Sirko et al., 2007). The DSD-1 positive cells form neurospheres in culture and demonstrate self-renewal properties (von Holst et al., 2006). Removal of CS chains with chondroitinase ABC treatment reduces their ability to form neurospheres, impairs cell proliferation and differentiation, suggesting the importance of CS chains in maintaining the neurogenesis. Similar to NG2, the effect is also mediated through FGF2 binding to CS-GAGs, which then induced neurosphere formation, proliferation of cells and their differentiation toward neuronal lineage (Sirko et al., 2010). Furthermore, the effect is specific toward FGF2 but not EGF. When sulphation on GAG chains is prevented using sodium chlorate, a reduction of cell proliferation in the neurospheres is observed (Akita et al., 2008) suggesting that sulphations on CS-GAGs are important for the process.

In addition to NG2 and DSD-1, other CSPGs including neurocan, phosphacan and neuroglycan C, have also been detected in the neurogenic areas, such as in the ventricular zone (Ida et al., 2006).

## Conclusion

CSPGs and HSPGs have widespread effects on many aspects of CNS development, function and ageing. Most of these effects depend on the sulphation of the GAG chains, which is determined by the action of many sulfotransferases and sulfatases. While GAG lysases and sulfatases have been explored as therapeutic agents, sulfotransferases have received little attention. Modulation of sulphation patterns through inhibition or activation of sulfotransferases is an attractive avenue for the development of new therapeutics for CNS diseases.

## Author Contributions

JF and JK participated equally in writing the review. Both authors contributed to the article and approved the submitted version.

## Conflict of Interest

The authors declare that the research was conducted in the absence of any commercial or financial relationships that could be construed as a potential conflict of interest.

## Publisher’s Note

All claims expressed in this article are solely those of the authors and do not necessarily represent those of their affiliated organizations, or those of the publisher, the editors and the reviewers. Any product that may be evaluated in this article, or claim that may be made by its manufacturer, is not guaranteed or endorsed by the publisher.
